# Role of hemoclips in the management of acute bleeding from a gastric stromal tumor: a case report and review of the literature

**DOI:** 10.1186/1752-1947-1-136

**Published:** 2007-11-14

**Authors:** Mouen A Khashab, Harvey M Cramer, Suthat Liangpunsakul

**Affiliations:** 1 Division of Gastroenterology and Hepatology, Department of Medicine and Department of Pathology and Laboratory Medicine, Indiana University School of Medicine, Indianapolis, IN 46202, USA

## Abstract

**Introduction:**

Though gastrointestinal stromal tumors (GISTs) frequently present with gastrointestinal bleeding, the guidelines for the management and control of bleeding are unclear especially in patients who are not appropriate for surgical resection.

**Case presentation:**

We report a case of gastric GIST in an elderly patient who presented with bleeding. Homeostasis was achieved initially with the endoscopic placement of a hemoclip followed by treatment with the tyrosine kinase inhibitor, imatinib.

**Conclusion:**

The management of bleeding GISTs in the elderly pose a challenging task to the gastroenterologist and treatment strategies should be tailored to the expertise of the endoscopist, surgeon and other supportive staff.

## Introduction

Mesenchymal tumors are an infrequently encountered group of benign and malignant neoplasms of the gastrointestinal tract. Gastrointestinal stromal tumors (GISTs), account for the majority of these gastrointestinal mesenchymal tumors. Mazur and Clark [1] first recognized GISTs as a separate entity from gastrointestinal smooth muscle tumors in 1983 based on the different cellular origin, the intestinal pacemaker cells of Cajal for the former, and the smooth muscle cells for the latter. GISTs are often asymptomatic and discovered incidentally during endoscopic procedures. GI-related symptoms normally occur with larger tumors. Several major GIST-related symptoms are bleeding, abdominal pain, abdominal mass and obstruction. When such complications occur, surgical resection is generally recommended. In this paper, we describe the case of an 84 year-old man who presented with melena and bleeding from a gastric GIST. Surgical intervention was prohibited due to other concomitant medical illnesses. However, the bleeding was successfully controlled with hemoclip application. The management of gastrointestinal bleeding from GISTs is also reviewed.

## Case presentation

**Figure 1:**
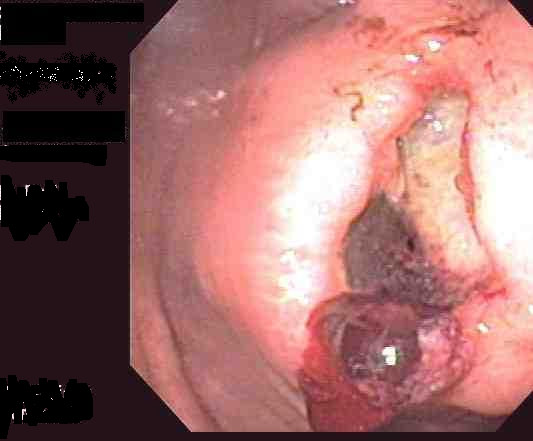
EGD showed the gastric mass with deep ulceration and bleeding visible vessels.

**Figure 2:**
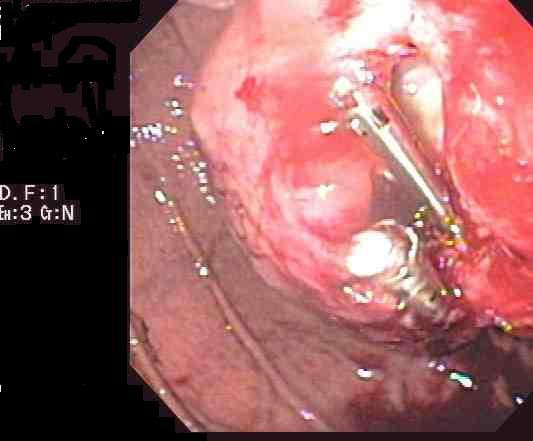
Homeostasis was achieved with placement of two hemoclips during upper endoscopy.

**Figure 3:**
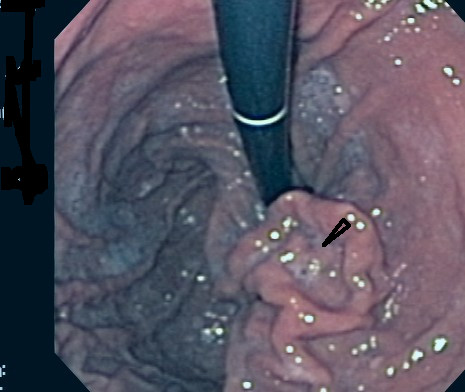
Repeat upper endoscopy 5 months after the episode of bleeding showed significant reduction of the tumor size and the previously identified ulcers were completely healed (arrow head).

An 84-year-old man had an incidental finding of a stomach mass on an abdominal CT scan performed in preparation for an inguinal hernia repair. Esophagogastroduodenoscopy (EGD) was performed and showed a gastric deformity suggestive of a subepithelial tumor rather than extrinsic compression. Endoscopic ultrasound (EUS) showed an intramural (subepithelial) lesion in the greater curvature of the stomach, 3 cm distal to the gastroesophageal junction measuring 44 mm × 59 mm. EUS-guided fine needle aspiration was performed and cytological examination showed spindle shaped cells that stained positive for c-kit. The cytomorphological and immunocytological findings were suggestive of a diagnosis of gastrointestinal stromal tumor (GIST). Shortly after the diagnosis, the patient presented to our emergency department with a one week history of epigastric pain, melena and dizziness. Upon examination, his temperature was 37.4°C, pulse 72 beats/min, respirations 18 times/min, and blood pressure 164/78 while lying down and 105/71 while standing. The patient was alert and oriented. Laboratory tests revealed the following values: hemoglobin 10.6 g/dl (normal 12–15) and hematocrit 31.5% (normal 35–49). His basic metabolic profile, coagulation profile and liver function tests were all normal. He was resuscitated with intravenous fluids and transfused with two units of packed red blood cells. Repeat EGD showed the GIST tumor with deep ulceration and visible bleeding vessels (Figure 1). Homeostasis was achieved with placement of two hemoclips (Figure 2). The patient was evaluated by the general surgery team and deemed not a good surgical candidate due to his age and severe coronary artery disease. He was started on imatinib (Gleevec; Novartis Pharmaceuticals Corporation, East Hanover, NJ) 400 mg daily by mouth in accordance with oncology recommendations. Patient was discharged home with no further gastrointestinal bleeding. Upon follow up five months later, the patient was continuing to do well with no further episodes of gastrointestinal bleeding. His follow up hemoglobin was 13.4 g/dL and hematocrit 39%. Repeat upper endoscopy showed significant reduction in the size of the tumor. The previously identified ulcers were completely healed (Figure 3, arrow head).

## Discussion

Approximately 2000–5000 cases of GIST are diagnosed annually. The predominant site for a GIST is the stomach (52%), followed by the small intestine (25%) [2]. The clinical presentation of a GIST largely depends on its size. Small tumors (usually measuring less than 2 cm) usually do not produce symptoms and are often detected incidentally on endoscopy or radiographic examination. Approximately one-third of patients present with GI bleeding. Bleeding can be occult and manifest as anemia, or overt and manifest as melena, hematochezia or hematemesis [3].

The standard therapy for GISTs regardless of presentation is complete surgical resection. In general, only a segmental resection of the organ in which the tumor originates is necessary. Meticulous surgical technique is essential as these tumors are fragile and tumor rupture increases the risk of peritoneal recurrence. Although complete resection is achieved in 85 % of patients, the 5-year survival after resection is approximately 50 % because of tumor recurrence [3,4]. Thus other treatment modalities in addition to surgical resection are needed to improve the treatment outcome. The discovery that dysregulation in the KIT tyrosine kinase activity underlies the pathogenesis of GIST has led to the development of a novel systemic tyrosine kinase inhibitor, imatinib[5]. This medication has revolutionized the treatment of this tumor particularly in patients who have metastatic and/or unresectable disease.

Currently, there are no guidelines on the management of patients with GIST presenting with acute upper GI bleeding. The initial evaluation includes an assessment of hemodynamic stability and resuscitation, if necessary. Therapeutic endoscopy is usually performed in cases of active GI bleeding. In this report, hemoclips were used and hemostasis was achieved. Because of the paucity of data on the management of actively bleeding GIST with hemoclips, we searched MEDLINE for literature published between January 1966 and February 2007 to compile the outcome of treating bleeding GISTs with hemoclips. The terms utilized in the search were gastrointestinal stromal tumor, gastrointestinal hemorrhage, bleeding, management, and clips/hemoclips. Reference lists of the identified articles were also reviewed to find additional cases. Although there are several reports of small intestinal and colonic GISTs presenting with acute GI bleeding, we identified only five case reports of six patients with stomach GIST presenting with acute GI bleeding [6-10]. One report of two patients is published in Italian [6]. Hemoclips were not used in any of these cases although there is a single case report of hemoclip use to control bleeding from an ulcerated duodenal GIST [11]. We report the first case of stomach GIST presenting with acute GI bleeding and treated with hemoclip application. The four cases that were published in English included three men and one woman with a mean age of 68.2 years (range, 50–77 years) [7-10] (Table 1). The clinical presentations of each patient are described in Table 1. No details regarding endoscopic interventions were provided; all patients underwent surgery to control the bleeding lesions.

**Table 1:** Clinical Data of four patients with stomach GIST presenting with acute GI bleeding

Year (reference)	Age (y)/sex	Clinical presentation	Treatment
2005 [7]	50/F	Recurrent upper GI bleed	Total gastrectomy, partial hepatectomy and esophagectomy
2005 [8]	72/M	Chest discomfort and syncope	Partial gastrectomy
2005 [9]	74/M	Hematemesis and melena	Partial gastrectomy
2006 [10]	72/M	Coffee ground vomiting	Partial gastrectomy

Since surgery was prohibited in our case due to other co-morbidities, imatinib was given after endoscopic treatment. The reason for this approach was twofold. First, this medication has proven efficacy in patients with metastatic disease who are usually treated non-surgically[12]. Second, it was given with the intent to cause regression of the size of the tumor and prevent future bleeding. In fact, upon repeating upper endoscopy three months afterwards, we found significant reduction in the size of the tumor and the ulcers were completely healed.

## Conclusion

In a clinical scenario when surgical resection is not an option in the management of bleeding gastric GIST, the use of hemoclips, if feasible, should be considered. The therapy should be followed by imatinib with the goal to stabilize the tumor growth and lead to regression of the size of the tumor. The management of bleeding gastrointestinal stromal tumors in the elderly poses a challenge to the gastroenterologist and treatment strategies should be tailored to the expertise of the endoscopists, surgeons, and other supportive staff members.

## Competing interests

The author(s) declare that they have no competing interests.

## Authors' contributions

The authors were involved in patient management or writing of the manuscript.

## Consent

Full verbal and written informed consent has been obtained from the patient for submission of this manuscript for publication.
